# Supplementary material for: Comparative multiomics analysis of cell physiological state after culture in a basket bioreactor
Source: Sci Rep. 2022 Nov 23;12:20161. doi: 10.1038/s41598-022-24687-4 (PMC9686226; doi:10.1038/s41598-022-24687-4)

70920934:+@NW_023666055.1:70938239:70938368:+@NW_023666055.1:70942019:70942254:+@NW_023666[illegible]

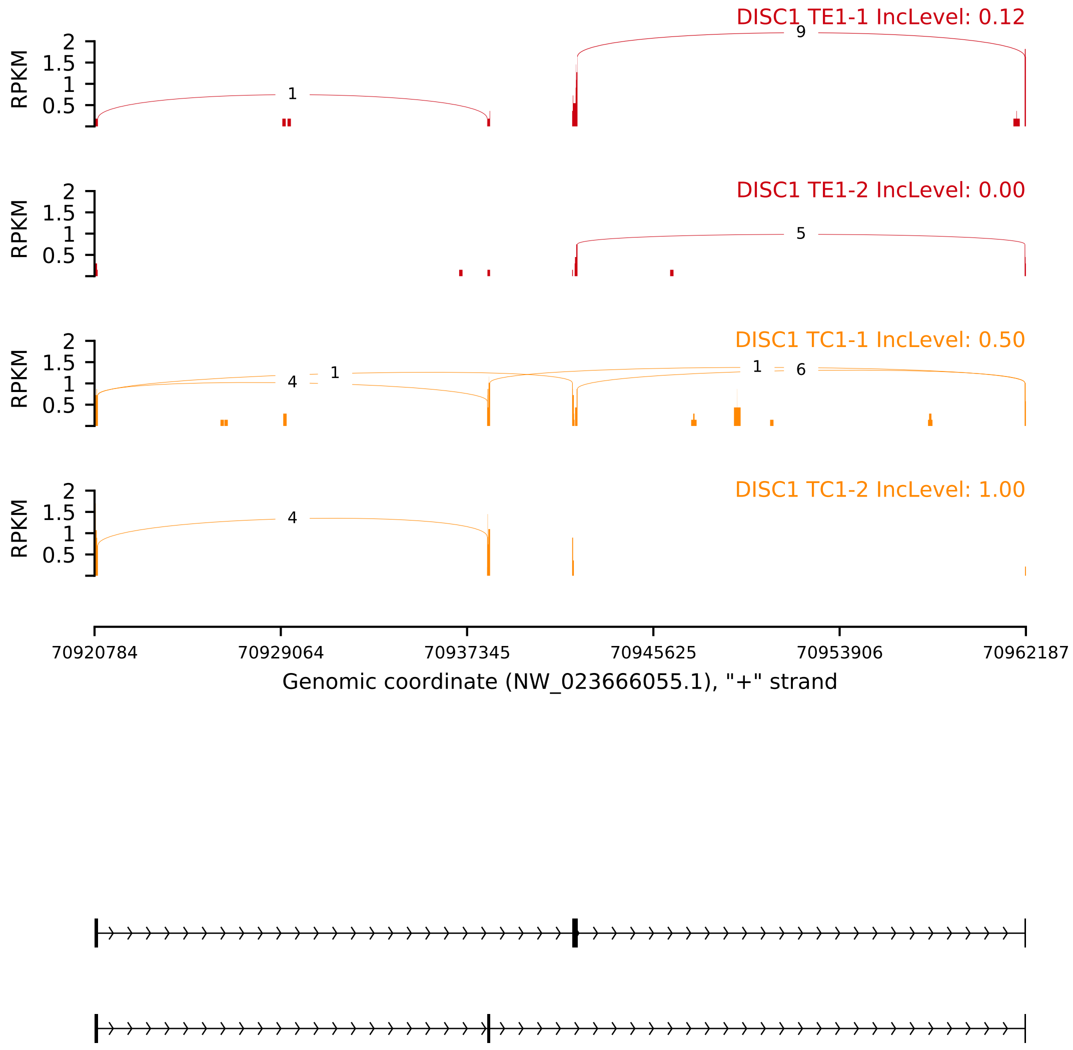

Supplement: Supplementary file 1 — Supplementary Information 1. [file 41598_2022_24687_MOESM1_ESM.zip › raw data/Transcriptomic raw data/7.AS/2.dasplot/TE1vsTC1/MXE/17_DISC1_NW_023666055.1_70920784_70920934_+@NW_023666055.1_70938239_70938368_+@NW_023666055.1_70942019_70942254_+@NW_023666055.1_70962134_70962188_+.pdf]
